# Supplementary material for: Molecular mechanism of allosteric activation of the CRISPR ribonuclease Csm6 by cyclic tetra-adenylate
Source: EMBO J. 2023 Dec 19;43(2):304–15. doi: 10.1038/s44318-023-00017-w (PMC10897365; doi:10.1038/s44318-023-00017-w)
Supplement: Supplementary file 1 — Appendix [file 44318_2023_17_MOESM1_ESM.docx]

**Appendix Figures**

**Molecular mechanism of CRISPR ribonuclease Csm6 allosterically activated by cyclic tetra-adenylate**

_Liyang Du, Qinwei Zhu and Zhonghui Lin_

| **Table of Contents** |  | **page** |
| --- | --- | --- |
| **Appendix Fig S1** | LC and MS analyses of cA_4_ cleavage by TtCsm6. | 2 |
| **Appendix Fig S2** | Sequence alignment of TtCsm6 CARF domain with the type III CRISPR standalone ring nucleases. | 3 |
| **Appendix Fig S3** | Analysis of TtCsm6 ribonuclease activity using denatured polyacrylamide gel electrophoresis. | 4 |
| **Appendix Fig S4** | HPLC analysis of cA_4_ cleavage in the MST binding assay. | 5 |
| **Appendix Fig S5** | Superposition of TtCsm6 structures in different states. | 6 |
| **Appendix Fig S6** | Catalytic site architecture of the TtCsm6 HEPN domain in the absence of cA_4_ | 7 |
| **Appendix Fig S7** | Structural Comparison of TtCsm6 with other type III CRISPR ancillary nucleases. | 8 |

**
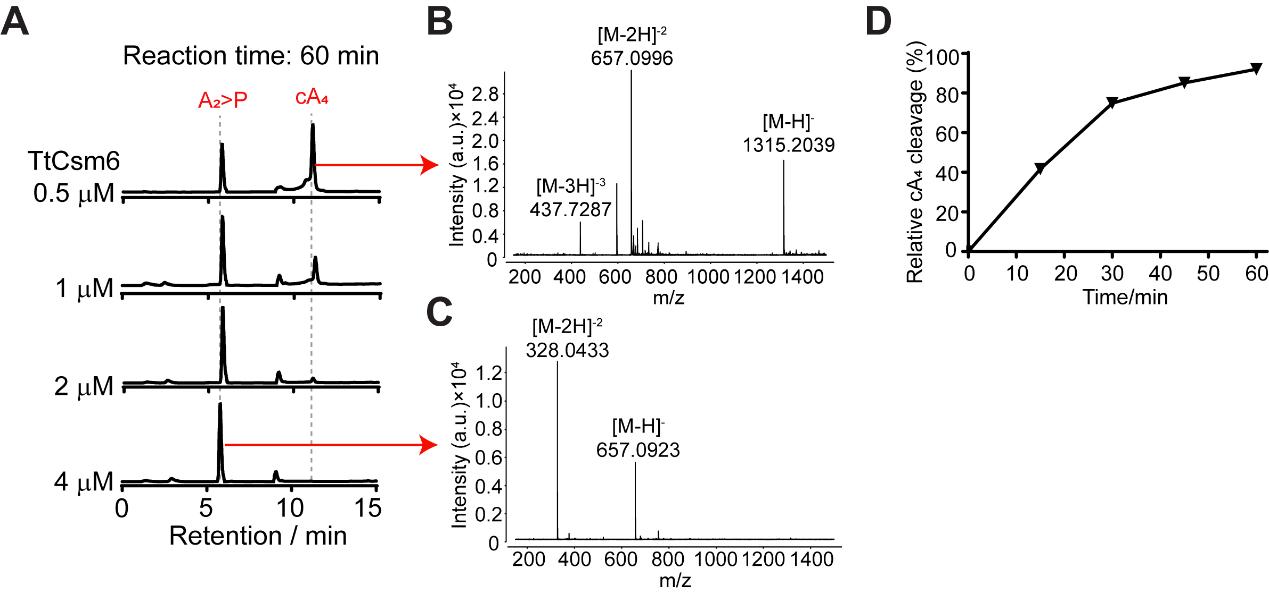
**

**Appendix Fig S1. LC and MS analyses of cA_4_ cleavage by TtCsm6.** **A.** HPLC spectra showing the reaction products of cA_4_ with increasing concentrations of TtCsm6. Reactions were carried out at 37°C for 60 min. **B, C.** Mass spectra of the samples eluted from HPLC column at specific retention times. Retention time 5.5-6.0: *m/z* 657.0923 for A_2_>P^-1^; *m/z* 328.0433 for A_2_>P^-2^; Retention time 11.0-11.5: *m/z*1315.2039 for cA_4_^-1^; *m/z* 657.0996 for cA_4_^-2^; *m/z* 437.7287 for cA_4_^-3^. **D.** The kinetic plot of cA_4_ cleavage with 2 μM TtCsm6 at 37°C. The reactions were monitored over a period ranging from 15 to 60 min, and each time point was tested once.

**
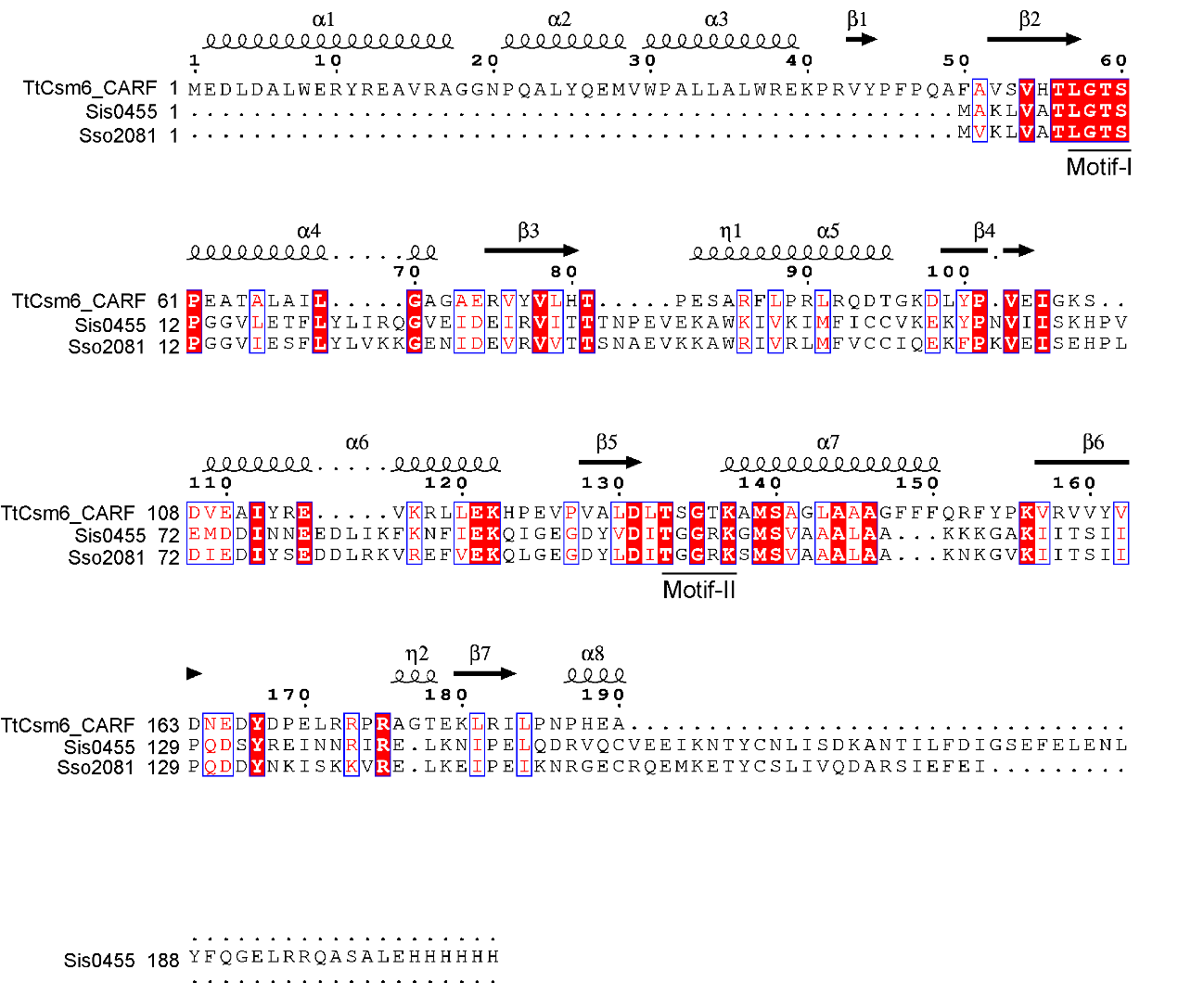
**

**Appendix Fig S2. Sequence alignment of TtCsm6 CARF domain with the type III CRISPR standalone ring nucleases.** The alignment was generated using the ESPript 3.0 server. Secondary structural elements of TtCsm6 are indicated above the sequences. Abbreviations: Sso, *Saccharolobus solfataricus*; Sis, *Sulfolobus islandicus*; Tt, *Thermus thermophilus.*

**
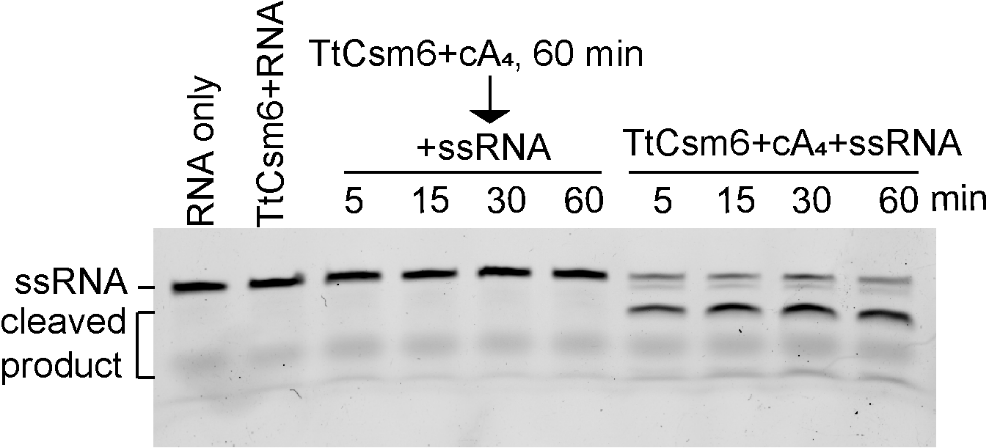
**

**Appendix Fig S3. Analysis of TtCsm6 ribonuclease activity using denatured polyacrylamide gel electrophoresis.** Reactions were carried out at 37℃ with 150 nM TtCsm6, using the FAM-labeled ssRNA as the substrate.

**
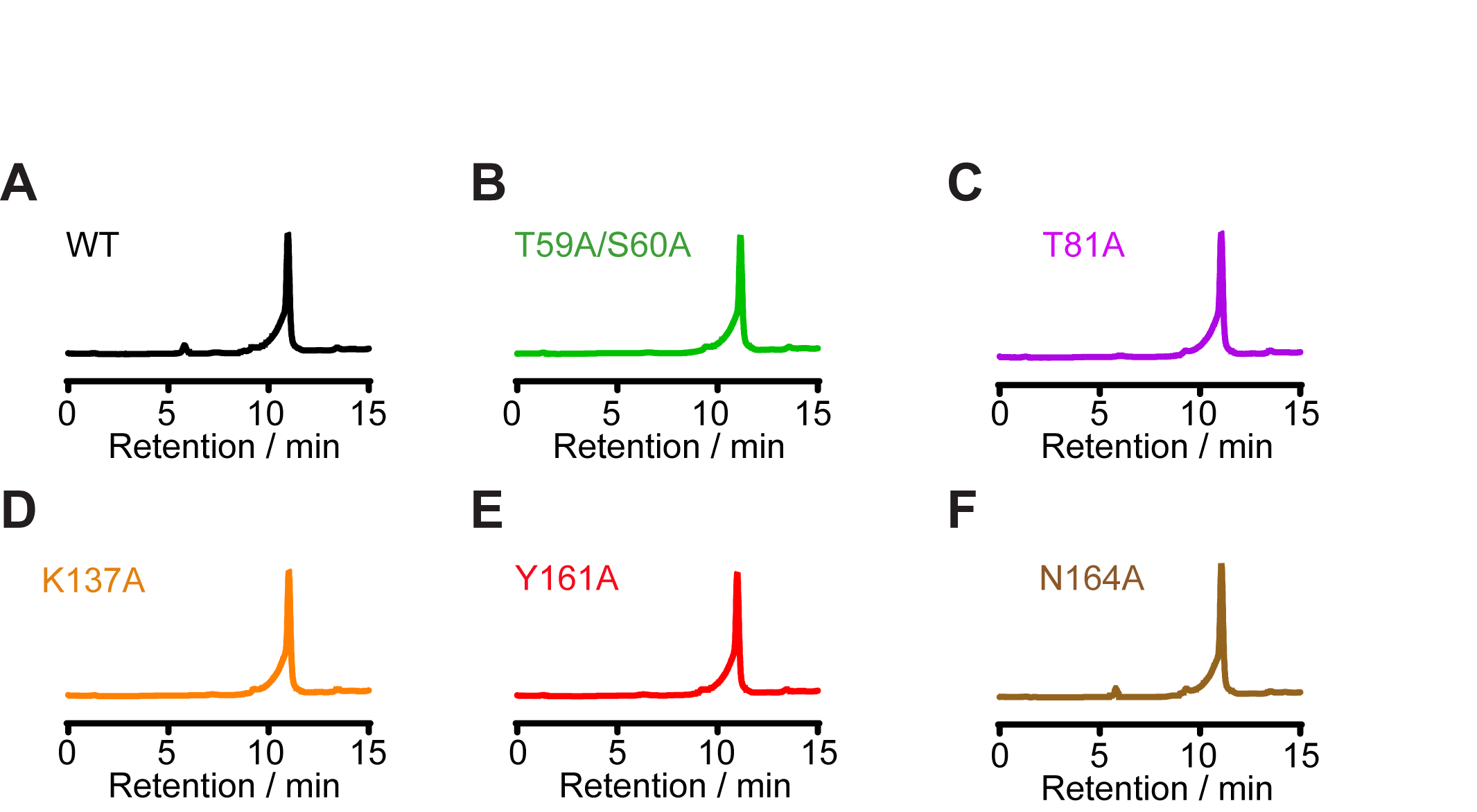
**

**Appendix Fig S4. HPLC analysis of cA_4_ cleavage in the MST binding assay.** 50 nM WT (**A**) or mutant (**B-F**) TtCsm6 protein was incubated with 4 µM cA_4_, at room temperature for 15 minutes in a phosphate buffered saline solution at pH 7.4, supplemented with 0.05% Tween-20.

**
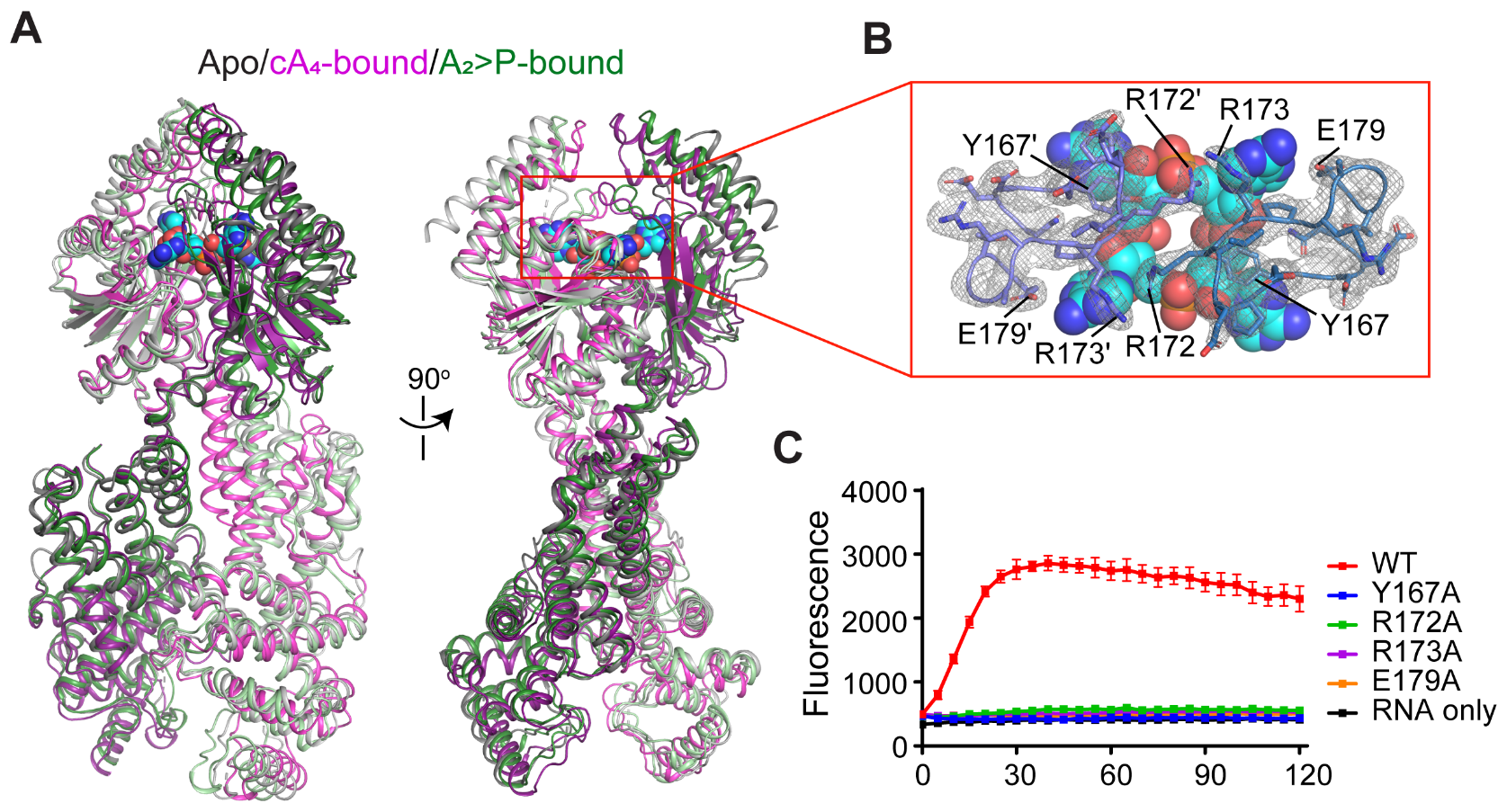
**

**Appendix Fig S5. Superposition of TtCsm6 structures in different states. A.** Structural alignment of apo-TtCsm6 (grey), cA_4_-bound TtCsm6 (magenta) and A_2_>P-bound TtCsm6 (Green). cA_4_ is shown in cyan sphere. **B.** Aarchitecture of the mobile loops in the cA_4_-bound CARF domain. The loops are superimposed with the 2Fo−Fc electron density map contoured at 1.0 σ. Critical residues are shown as sticks. **C.** The impact of mutation in the mobile loop on the ribonuclease activity for TtCsm6. Values are means ± SD, n = 3 for technical replicates.

**
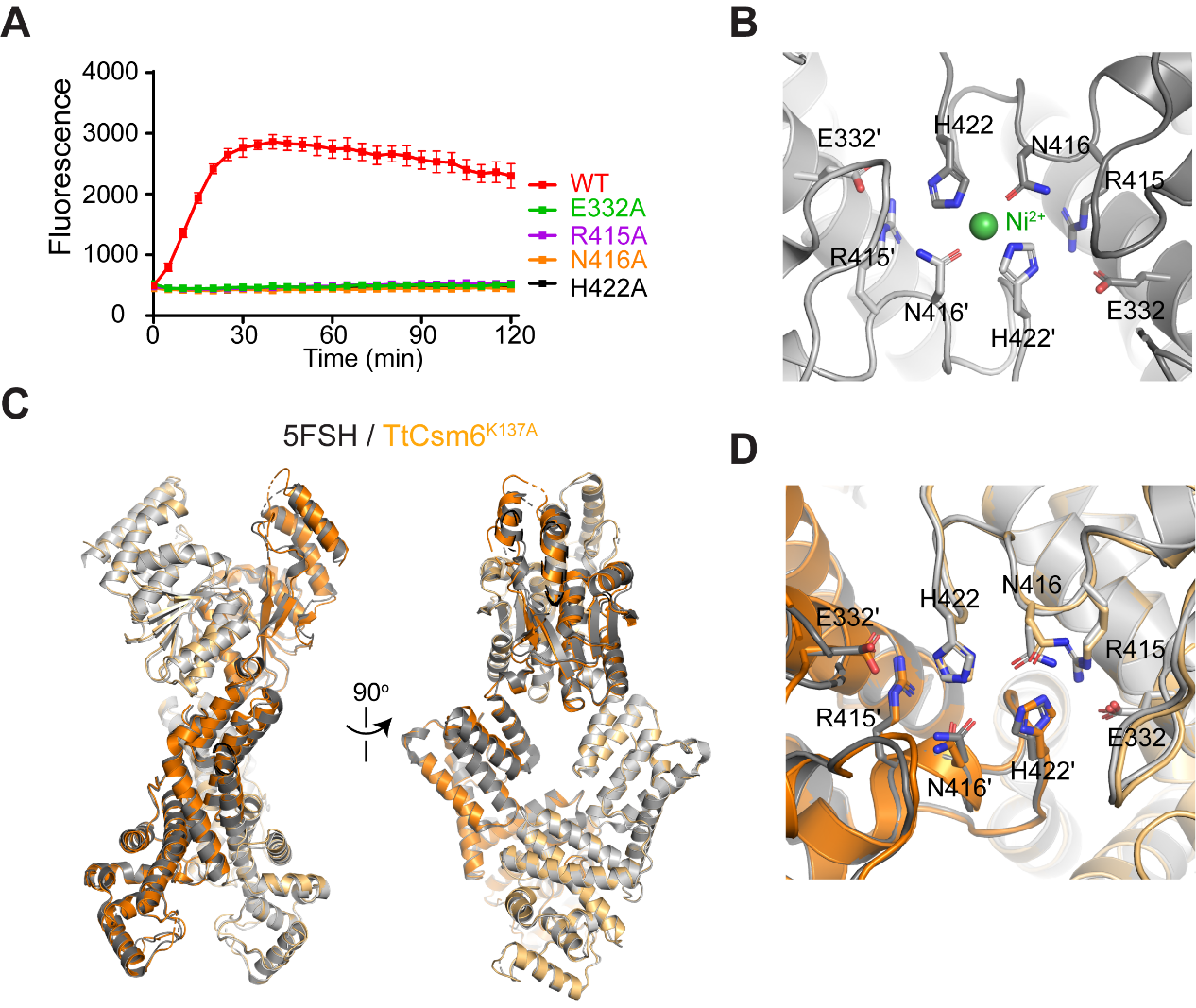
**

**Appendix Fig S6. Catalytic site architecture of the TtCsm6 HEPN domain in the absence of cA_4_. A.** Effect of point mutation within the R-X_4-6_-H motif of HEPN domain on the ribonuclease activity of TtCsm6. Values are means ± SD, n = 3 for technical replicates. **B.** Conformation of the R-X_4-6_-H motif in the published structure of TtCsm6 (PDB 5FSH). Key residues are shown in sticks. Ni^2+^ ion is represented as green sphere. **C.** Structural comparison between TtCsm6^K137A^ and the previous TtCsm6 (PDB 5FSH). **D.** Comparison of the conformation of R-X_4-6_-H motif between TtCsm6^K137A^ (orange) and 5FSH (grey).

**
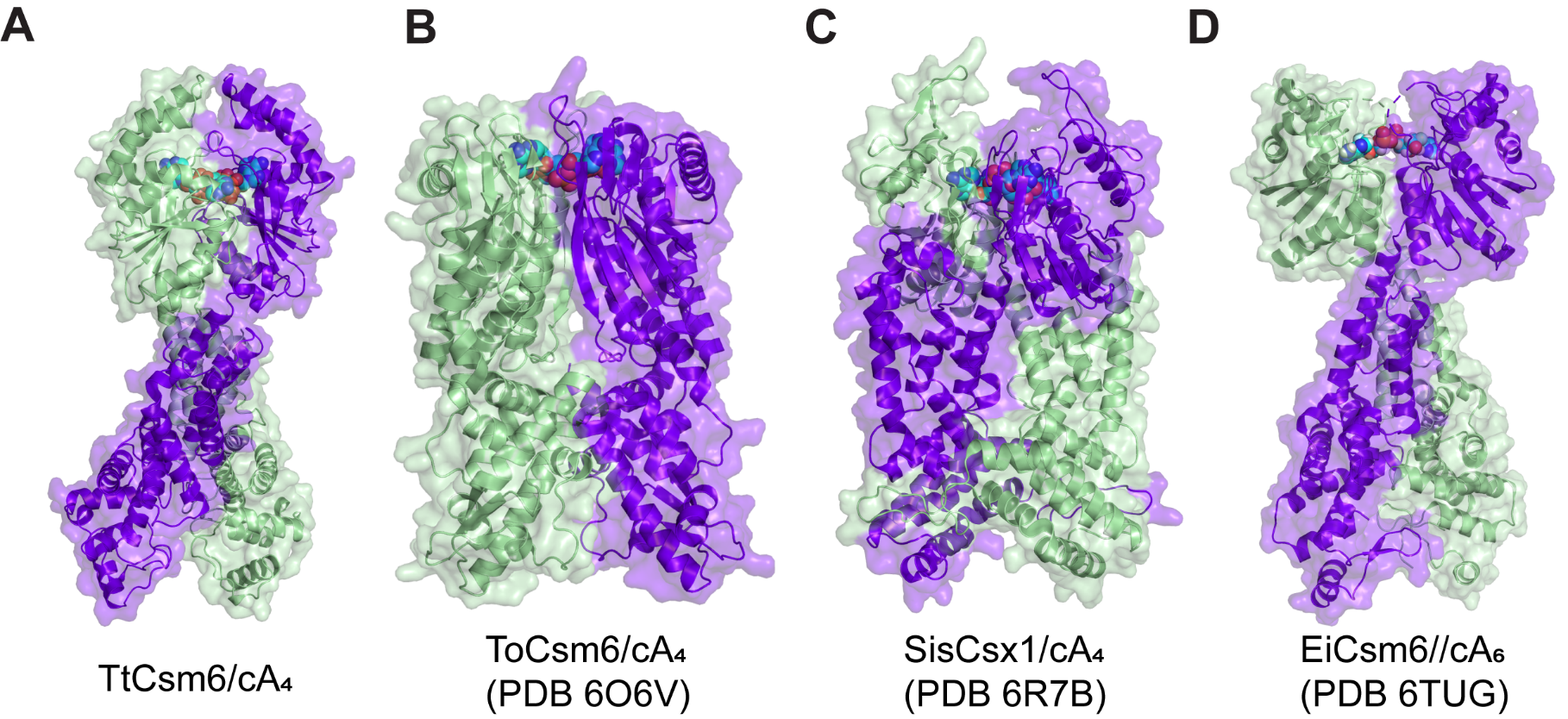
**

**Appendix Fig S7. Structural Comparison of TtCsm6 with other type III CRISPR ancillary nucleases.** The two monomers within the homo-dimer are colored in purple and green, respectively. cA_4_ is shown in cyan sphere. Tt, *Thermus thermophilus*; To, *Thermococcus onnurineus*; Ei, *Enteroccocus italicus*; Sis, *Sulfolobus islandicus.*
